# The association of family functioning, parenting knowledge, parenting behaviors, and family characteristics with early child growth: a study in a rural community of Southern Thailand

**DOI:** 10.3389/fpubh.2026.1757048

**Published:** 2026-03-19

**Authors:** Chutarat Sathirapanya, Chanon Kongkamol, Napakkawat Buathong, Pornchai Sathirapanya, Chuphanat Ananthaninkoon, Dittakorn Atthakornpreeda, Panthita Chanthin, Pornlapas Suksubwasin, Laphat Phanawetsanti, Wattanachai Chasuwan, Apiwut Tantiwiwattkul

**Affiliations:** 1 Department of Family and Preventive Medicine, Faculty of Medicine, Prince of Songkla University, Hat Yai, Songkhla, Thailand; 2 Research Center for Kids and Youth Development, Prince of Songkla University, Hat Yai, Songkhla, Thailand; 3 Department of Medicine, Faculty of Medicine, Prince of Songkla University, Hat Yai, Songkhla, Thailand

**Keywords:** behaviors, early childhood, family functioning, growth, knowledge, parents

## Abstract

**Objective:**

This study aimed to evaluate the association of family functioning (FF), parenting knowledge (PK), parenting behaviors (PBs), and family characteristics (FCs) with child growth.

**Methods:**

This cross-sectional study was conducted in a community in Southern Thailand. Parents or childcare providers (CPs) who had cared for the children aged 2–6 years for ≥ 6 months were invited to join the study voluntarily. FF, PK, PBs, and FCs were evaluated. Their associations with child growth were assessed using multiple logistic regression, adjusting for all variables in the R program (*p* < 0.05).

**Results:**

A total of 102 parents or CPs who consented to participate were enrolled. Low FF score (adjusted odds ratio (adj OR) = 5.11 (1.57,18.60), *p* = 0.006) and male caregivers (adj OR = 6.13 (0.88,47.80), *p* = 0.027) were significantly associated with unfavorable PBs. In the logistic regression model, inadequate PK (adj OR = 0.14 (0.02,0.71), *p* = 0.016), unfavorable PB (adj OR = 0.10 (0.01,0.72), *p* = 0.021), caregivers’ age (adj OR = 3.14 (0.52,22.70) 32–37 years; and (adj OR = 24.30 (2.73,37.7) 38–45 years, *p* = 0.001), and marital status (adj OR = 15.80 (2.13, 16.50), *p* = 0.006) were associated with height, while parental status (parent vs. non-parent) was associated with weight (adj OR = 0.43 (1.14, 29.4), *p* = 0.030).

**Conclusion:**

Child growth was significantly affected by the integrative effects of parental and familial factors, not limited to FF.

## Highlights

What are the main findings?

- Parenting knowledge, parenting behaviors, age, and marital status of the parents or childcare providers (CPs) were significantly associated with children’s height, while parental status (parent vs. non-parent) was significantly associated with weight.
- Family functioning score, parents’ education, sex, parental status, and family structure were associated with parenting behaviors.
- Family functioning score was not associated with the children’s weight and height.

What are the implications of the main findings?

- Family functioning is not the sole factor affecting child growth during early childhood.
- Comprehensive interventions integrating parental and family factors are essential for promoting early childhood growth and development.

## 1 Introduction

Facilitating growth and development during early childhood is crucial for a healthy adult life. Every parent or childcare provider (CP) plays a key role for early childhood care. The integration of healthy family functioning (FF), good parenting knowledge (PK), favorable parenting behaviors (PBs), and familial characteristics (FCs) interactively influences children’s wellbeing and daily living. Among them, FF has been suggested as a principal modulator of the interrelations (1–3).

FF is commonly described as the interpersonal, behavioral, and psychological relationships that regulate family life within a family context (4, 5). FF encompasses family dynamics such as emotional bonds, closeness of relationships, positive feedback or communication, and compliance with family rules, which collectively lead to efficient management of family routines and problem-solving (6, 7). Thus, FF can be regarded as an influential mediator of happy family living (3, 8).

Although PBs represent the practical aspects of child rearing, FF, PK, PBs, and FCs were interrelated and jointly influence them. PBs, as well as FF, can be shaped or modified as parents acquire greater knowledge, understanding, and experience in raising a child. One study demonstrated that by understanding the triggers of unhealthy dietary behaviors in children, knowledgeable parents could provide reasoned feedback and apply gentle behavioral interventions based on FF and PBs principles to deal with the issue. These studies emphasized the additive effects of FF and PBs when the PK of the child’s eating habits was accounted (9, 10). Another study also reported the association of parental knowledge and attitudes with adequate intake of high-nutrition index meals among preschool children (11). Therefore, parents, or CPs in our study, were the facilitators of healthy eating behaviors of children, leading to healthy outcomes. The success needed a proper integration of the related parental and family factors.

**Figure 1:**
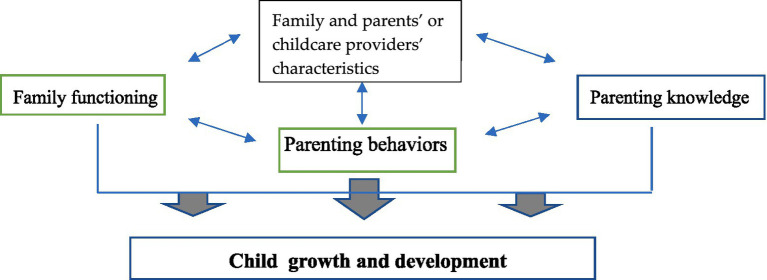
Conceptual framework of the association of family functioning, parenting knowledge, and parenting behaviors with child growth and development.

As the eating habits and nutritional status among early childhood or preschool children (<7 years) depend absolutely on their parents’ or CPs’ feeding. Acquiring accurate PK about child nurturing, applying friendly PBs, and healthy FF in managing the children’s eating habits are mandatory for a healthy diet and nurturing among this group of children. Until now, there have been limited studies demonstrating the effects of parental and family characteristics on early childhood growth and development. This study aimed to investigate the association of these factors with child growth and development among preschool children in a rural community of Southern Thailand. The percentage of appropriate growth and development among the early childhood children in this community, a district of a lower-southern province of Thailand, was at or higher than the national record (12). We aimed to explore the background of PK, PBs, the role of FF, and FCs that promoted child growth and development in this community. We expect that the findings of this study will inform the design of future health plans at promoting child growth and development. The proposed conceptual framework for this study is shown in Figure 1.

## 2 Materials and methods

### 2.1 Study design and setting

This research was a cross-sectional study that aimed to describe the associations of FF, PK, PBs, and FCs with child growth among preschool children aged 2–6 years in a subdistrict of a southern province of Thailand. The study area is an island in the Songkhla Lake located 6 km away from the mainland by road. With an area of 164 km^2^, it comprises 2,623 households. The total population of the subdistrict during the study period was 7,742, of whom 3,761 were male and 3,981 were female. Nearly 88% of the population practiced Buddhism, while the remainder practiced Islam. Fishing and rubber farming were the primary occupations of the local people. There were two child daycare centers (CDCs) and one subdistrict health promotion hospital (SHPH) in the study area. The total number of preschool-aged children listed in the community was 138 (13).

### 2.2 Study population, and inclusion and exclusion criteria

One of the parents, or CPs, who had provided care for the children for at least 6 months were invited to participate in this study after the study information was provided. Participants were proficient in both spoken and written Thai. Their participations were voluntary.

### 2.3 Sample size and enrollment

The required sample size for significant statistical power in this study was calculated by G*power software, where alpha was set at 0.05 (Type I error), power at 0.95, and effect size at −0.3. A sample size of 134 (121 plus 10% for missing cases) was required.

### 2.4 Terms and definitions

- Early childhood was defined as children aged < 7 years, which is considered the golden period for facilitating child growth and development.
- Family functioning (FF) was the family dynamics that resulted from emotional and behavioral interactions among family members. The process comprises emotional status or positive relationships, reasoning, communication or discussion, and rational manipulation for solving internal and external conflicts (1, 6).
- Parenting behaviors (PBs) were the parental skills of nurturing children based on correct child nurturing knowledge.
- Childcare provider (CP) was the person who cared for the children when their parents were away from home to make a living in agricultural fields or offices. The CP in this study was usually female and one of the children’s close relatives, such as a grandmother or aunt.
- Parenting knowledge included parents’ or CPs’ correct knowledge of child-rearing techniques aiming at the appropriate development of physical, emotional, and social relations.
- Family characteristics were the types of family structure, that is, nuclear family or extended family with more than one generation, either skipped or succeeding generations of family members lived together.

### 2.5 Study tools

Three questionnaires were used for data collection and evaluation:

1. The Family State and Family Functioning Assessment Scale (FSFAS) is a short assessment tool for evaluating FF. The test had 25 questions to assess the 5 domains of FF, that is, family support (questions 1–5), family discipline (questions 6–11), positive communication and problem solving (questions 12–16), emotional status (questions 17–21), and parent–child relationship (closeness) (questions 22–25). The answer for each question was divided into four levels of the family’s state and function perceived by the study participants: strongly disagree, disagree, moderately agree, and strongly agree.

a. For questions 1–16, “strongly disagree” was given 1 point, “disagree” 2 points, “moderately agree” 3 points, and “strongly agree” 4 points.

b. For questions 17–25, “strongly disagree” was given 4 points, “disagree” 3 points, “moderately agree” 2 points, and “strongly agree” 1 point.

The levels of FF were determined by classifying the sum of the scores obtained from each question into three levels: low, moderate, and high. The score ranges for the classification of levels were obtained by subtracting the lowest score from the highest score and dividing by 3 (100–25/3 = 25). Therefore, scores of 25–50 were low, while 51–75 and 76–100 were moderate and high levels of family functioning, respectively. Content reliability of the FSFAS, tested using Cronbach’s alpha, was 0.87 for the total form and 0.70–0.84 for the individual questions documented in the validation study of the questionnaire in Thailand (14).

2. Parenting knowledge (PK): The section evaluated the parents’ or CP’s correct understanding of childcare, which was a part of the Early Childhood Care Literacy Assessment developed by the Department of Health, Ministry of Public Health, Thailand (15). The content validity of the questionnaire was 0.93, and its reliability was 0.94. The questionnaire consisted of 15 yes–no answer questions. The questions included five domains: physical development (three questions), emotional control (three questions), social skills (three questions), intellectual development (three questions), and environmental management (three questions).

The scoring method was based on the correct answer to each question, for which 1 point was given for each correct answer. Additionally, 1 reward point was given for the answer “yes” to questions 1, 3–5, 9–10, 12, 14, or 15, and “no” to questions 2, 6–8, 11, or 13. A score from 12 to 15 points was evaluated as having adequate knowledge, while 9 to 11 points was considered fair, and below 9 points was considered inadequate PK (15).

3. Parenting behaviors (PBs): The Parenting Behavior Assessment Questionnaire was used to evaluate PBs in this study. The questionnaire was composed of three domains: nurture and support (questions 1–8), positive discipline (questions 9–16), and child development promotion (questions 17–24). The answers were based on the estimated frequency (times/week or month) of doing the behavior, i.e., “regularly” was five or more/week (5 points), “often” three to four/week (4 points), “sometimes” one to two/week (3 points), “rarely” one to two times a month (2 points), and “never” no such behavior had ever been performed (1 point). The evaluation was based on the summation of the score of each question, where a score ≥ 96 indicated a favorable level of PBs in caring for early childhood. The questionnaire was tested for content validity by three experts specialized in the field of child and adolescent psychiatry and psychology. The validity tested was 0.6–1.0, and the reliability was 0.73–0.80 (16).

### 2.6 Data collection

Initially, we identified the children aged 2–6 years from the children’s name list of the local CDCs and SHPH. Either parent or a CP of the selected children, who fulfilled the inclusion criteria, was informed about the study details and verbally invited to participate in this study. Their decision to participate was voluntary. After the written informed consents for participation were signed, we interviewed and recorded the participants’ data when they brought their children to the CDCs and through home visits. The collected data were general demographics and FCs, that is, sex, age, marital status, educational level, occupation, family income, financial status, relationship with the children under their care (parental status), family structure, evaluation of PBs, PK, and FF. The data on children’s physical growth and development were retrieved from the records of the CDCs and SHPH.

### 2.7 Data analysis

Descriptive statistics were used for data analysis in this study. Frequency (%), mean (SD), or median (Q1, Q3) were used where appropriate. Chi-squared test or Fisher’s exact test was used to compare the significant differences, and adjusted ORs with all variables were presented to test the associations between variables (*p* < 0.05). All analyses were performed using the R program version 4.1.0.

### 2.8 Ethical approval

This study protocol was approved by the Ethics Committee of the Faculty of Medicine, Prince of Songkla University. (EC code: REC No.67–409–9-2, date of approval: 4 September 2024). We confirmed that the study was conducted in accordance with the principles outlined in the Declaration of Helsinki (1975, revised in 2013). Signed informed consent forms were obtained before starting the research. All the identifiable information of the study participants was completely anonymous. Data analysis was performed aggregately to ensure the participants’ confidentiality.

## 3 Results

### 3.1 General characteristics of the participants

Of the 138 parents or CPs who were invited, 102 participated in this study. Among these participants, 72 (70.6%) were the child’s parents, and 91 (98.2%) were female. Expanded family structures comprising three generations were observed in 48% of households. Among the participants, 44.1% had graduated from high school or vocational school. Monthly earnings ranged from THB 3,000 to 49,000, with 51% reporting insufficient income to meet essential living costs, and 35.3% were in debt (Table 1).

**Table 1:** General characteristics of the study participants (*n* = 102).

Characteristics	Total (*n* = 102) *n* (%)
Sex	
Female	91 (89.2)
Male	11 (10.8)
Age (years)	
Mean (SD)	38.5 (11.5)
Median (Q1, Q3)	37.0 (31.0,45.0)
Occupation	
Employees	27 (26.5)
Agriculture/Fishermen	31 (30.4)
Self-Employed /Business Owners	12 (11.8)
Unemployed / housewife	32 (31.4)
Educational level	
Bachelor’s or higher	28 (27.5)
Elementary school	29 (28.4)
High School/vocational Certificate	45 (44.1)
Family Income (Baht/Month)	
Mean (SD)	12,300 (82.1)
Median (Q1, Q3)	10,000 (7,280, 15,000)
Financial Status	
Inadequate with debt	36 (35.3)
Inadequate without debt	16 (15.7)
Adequate and with savings	20 (19.6)
Adequate but no savings	30 (29.4)
Marital Status	
Married	83 (81.4)
Single/widow/divorced	19 (18.6)
Parental status	
Not parents	30 (29.4)
Parents	72 (70.6)
Family structure types	
Nuclear family /single-parent	43 (42.2)
Skipped-generation	10 (9.8)
Three-generation	49 (48.0)

### 3.2 Family state and functioning

Based on the median scores achieved by the FSFAS-25 tool, 69 (67.6%) participants had high levels, while the remaining had medium to low levels (Table 2). Family discipline had the largest difference in median scores between the two groups (Table 2).

**Table 2:** Comparison of median (Q1, Q3) scores of individual domains of family functioning evaluated by FSFAS-25, parenting knowledge and parenting behaviors between groups (*n* = 102).

Family functioning	Median (Q1, Q3)		Total (*n* = 102)	*p-value*
	High (*n* = 69, 67.6%)	Low-Med (*n* = 33, 32.4%)		
Family support	19 (18.0, 20.0)	17.5 (15.0, 19.0)	19 (16.0, 19.0)	<0.001*
Family discipline	22 (20.0, 23.0)	10 (16.0, 20.0)	20 (18.0, 22.0)	<0.001*
Positive communication	18 (17.0, 20.0)	10 (15.0, 18.0)	18 (16.0, 19.0)	<0.001*
Emotional status	16 (15.0, 17.3)	10 (9.0, 13.0)	14 (11.0, 16.0)	<0.001*
Parent (or care provider) - child relationship	15 (14.0, 16.0)	10 (8.0, 12.0)	14 (10.0, 15.0)	<0.001*

Parenting knowledge	Fair-adequate (*n* = 43, 42.1%)	Inadequate (*n* = 59, 57.9%)	Total (*n* = 102)	*p-value*
Physical growth	2 (2.0, 3.0)	2 (1.0, 2.0)	2 (1.2, 2.0)	<0.001*
Emotional control	3 (3.0, 3.0)	3 (2.0, 3.0)	3 (2.0, 3.0)	0.00105*
Social skills	2 (2.0, 3.0)	1 (0.0, 1.0)	1 (1.0, 2.0)	<0.001*
Intelligence development	2 (2.0, 3.0)	2 (2.0, 2.5)	2 (2.0, 3.0)	0.0133*
Environmental management	3 (3.0, 3.0)	3 (2.0, 3.0)	3 (3.0, 3.0)	<0.001*

Parenting behaviors	Favorable (*n* = 74, 72.5%)	Unfavorable (*n* = 28, 27.5%)	Total (*n* = 102)	*p-value*
Nurture and support	38 (36.0, 39.0)	29 (27.0, 34.0)	36 (34.0, 39.0)	<0.001*
Positive discipline	32 (30.0, 34.8)	27 (25.0, 28.0)	31(27.0, 34.0)	<0.001*
Promotion of child development	36 (34.0, 38.0)	30 (25.8, 31.0)	34.5(31.0, 37.0)	<0.001*

* < p = 0.05, Mann–Whitney U Test.

### 3.3 Parenting knowledge

We found that 43 (42.1%) participants had a fair to adequate level of PK in childcare. Considering the individual domains of PK evaluated, only social skills showed a difference in median scores between the groups.

### 3.4 Parenting behaviors

We found that 74 (72.5%) participants had favorable levels for PBs. Nurture and support showed the largest difference in median scores between the two groups (Table 2).

All the individual domains of FSFAS, PK, and PBs showed significant differences between the two groups of comparison of each questionnaire (Table 2).

### 3.5 Associations of FF, PK, PBs, and FCs with child height and weight

The study found that FF, as well as sex, educational level, parental status, and family structure type, were significantly associated with PBs (Table 3). Furthermore, PBs, PK, age group, and marital status were significantly associated with child height after adjusted with all variables (Table 4). However, only parental status was significantly associated with child weight (*p* = 0.030) (Table 5). The child development data were inadequate for statistical analysis. Therefore, the results of child development in this study were unavailable.

**Table 3:** The associations of family functioning, parenting knowledge, and family characteristics with parenting behaviors (adjusted odds ratio with all variables) (*n* = 102).

Variables	OR	95% CI	*p*-value	Adj OR	95% CI	*p*-value
FSFAS						0.006*
High	—	—		—	—	
Low	2.89	1.17, 7.25	0.021	5.11	1.57,18.6	
Sex						0.027*
Female	—	—		—	—	
Male	2.46	0.66, 8.95	0.20	6.13	0.88, 47.8	
Education level						0.005*
Elementary	—	—		—	—	
High school/Vocational	0.39	0.14, 1.03	0.060	0.33	0.08, 1.23	
Bachelor’s and higher	0.08	0.01, 0.35	0.002	0.09	0.01, 0.56	
Parental status						0.010*
Not parents	—	—		—	—	
Parents	0.34	0.14, 0.86	0.023	0.13	0.02, 0.63	
Family structure type						0.001*
Nuclear family/Single parent	—	—		—	—	
Skipped-generation	0.66	0.13, 2.72	0.6	0.03	0.00, 0.27	
Three-generation	0.30	0.11, 0.77	0.015	0.11	0.01, 0.50	

FAFAS, The Family State and Family Functioning Assessment Scale; CI, confidence interval; OR, crude odds ratio. Adj OR, adjusted odds ratio, *p < 0.05. The variables with blank values (−) were references.

**Table 4:** The associations of family functioning, parenting knowledge, parenting behaviors, and family characteristics with the children’s height (adjusted odds ratio with all variables) (*n* = 102).

Variables	OR	95% CI	*p*-value	adj OR	95% CI	*p*-value
FSFAS						0.084
High	—	—		—	—	
Low	1.15	0.43, 2.90	0.8	4.70	0.81, 33.5	
Parenting knowledge						0.016*
Fair-adequate	—	—		—	—	
Inadequate	0.80	0.33, 1.99	0.6	0.14	0.02, 0.71	
Parenting behaviors						0.021*
Favorable	—	—		—	—	
Unfavorable	0.16	0.02, 0.60	0.018	0.10	0.01, 0.72	
Sex						
Female	—	—				
Male	1.11	0.23, 4.21	0.9			
Age group						0.001*
14–31	—	—		—	—	
32–37	1.27	0.38, 4.46	0.7	3.14	0.52, 22.7	
38–45	2.63	0.76, 9.63	0.13	24.3	2.73, 377	
46–72	0.50	0.10, 2.16	0.4	0.00		
Occupation						
Unemployed/housewife	—	—				
Fishing/agriculture	1.46	0.47, 4.72	0.5			
Employee/laborer	1.25	0.37, 4.23	0.7			
Self-employed/business owner	1.19	0.22, 5.38	0.8			
Education Level						
Elementary	—	—				
High school/vocational	0.79	0.26, 2.48	0.7			
Bachelor’s and higher	1.75	0.56, 5.70	0.3			
Salary	1.00	1.00, 1.00	0.5			
Marital status						0.006*
Married	—	—		—	—	
Single/ widow/divorced	2.63	0.90, 7.51	0.071	15.8	2.13, 165	
Parental status						
Not parents	—	—				
Parents	1.18	0.45, 3.36	0.7			

FAFAS, The Family State and Family Functioning Assessment Scale; CI, confidence interval; OR, crude odds ratio, adj OR-adjusted odds ratio.

*p < 0.05. The variables with blank values (−) were references.

**Table 5:** The associations of family functioning, parenting knowledge, parenting behaviors, and family characteristics with the children’s weight (adjusted odds ratio with all variables) (*n* = 102).

Variables	OR	95% CI	*p*-value	adj OR	95% CI	*p*-value
FSFAS						0.084
High	—	—				
Low	0.74	0.24, 2.02	0.6			
Parenting knowledge						
Fair-adequate	—	—				
Inadequate	1.07	0.41, 2.86	0.9			
Parenting behaviors						0.054
Favorable	—	—		—	—	
Unfavorable	0.21	0.03, 0.79	0.044	0.26	0.04, 1.02	
Sex						
Female	—	—				
Male	1.42	0.29, 5.46	0.6			
Age group						
14–31	—	—				
32–37	0.87	0.26, 2.95	0.8			
38–45	1.14	0.31, 4.15	0.8			
46–72	0.26	0.04, 1.22	0.12			
Occupation						
Unemployed/housewife	—	—				
Fishing/agriculture	1.24	0.39, 4.07	0.7			
Employee/laborer	0.62	0.15, 2.34	0.5			
Self-employed/business owner	1.19	0.22, 5.38	0.8			
Education level						
Elementary	—	—				
High school/vocational	1.37	0.43, 4.87	0.6			
Bachelor’s and higher	1.60	0.44, 6.13	0.5			
Marital status						
Married	—	—				
Single/ widow/divorced	1.39	0.40, 4.21	0.6			
Parental status						0.030*
Not parents	—	—		—	—	
Parents	5.38	1.43, 35.3	0.030	4.43	1.14, 29.4	
Family structure type						
Nuclear /single-parent	—	—				
Skipped-generation	0.37	0.02, 2.32	0.4			
Three-generation	0.96	0.36, 2.57	>0.9			

FAFAS, The Family State and Family Functioning Assessment Scale; CI, confidence interval; OR, crude odds ratio, adj OR-adjusted odds ratio.

*p < 0.05. The variables with blank values (−) were references.

## 4 Discussion

The effective nurturing of a child is influenced by multiple family factors and processes. The results of this study showed that children’s height was associated with PBs, PK, age group, and marital status of the parents or CPs (Table 4). For the children’s weight, only parental status of caregivers (parents vs. non-parents) was significantly associated, while PBs were marginally associated (*p* = 0.054) (Table 5). Given that children’s height and weight represented the outcomes of preschool children’s nurturing, parenting factors, except for FF, showed a significant association. A cross-sectional study by Walton et al. examined the association between parenting practices and nutrition risks in preschool children, as well as whether FF modified or confounded this association. The study found that using positive encouragement rather than restrictive parenting behaviors on children’s eating lowered the risks, while FF did not modify the association (5). However, FF, together with sex, educational level, parental status, and family structure, were associated with PBs in this study (Table 3). Hence, parenting and family characteristics can directly or indirectly impact the outcomes of child nurturing. The corporate interaction and impacts of the variables on the child’s growth and development depend on the individual socio-economic and cultural context of the family.

Theoretically, FF has been recognized as a significant facilitator of cognitive-based behavioral interactions among the family members, targeting at happy family life. Healthy FF could promote physical and psychological wellbeing in a family (17, 18). Based on this principle, FF has been further applied to modulate healthy eating behaviors among older children to prevent overweight and obesity (4, 6, 19–21). However, its implication on early childhood or preschool children to promote healthy eating resulting in appropriate growth and development are very limited. Since feeding the early childhood depends absolutely on parents’ or CPs’ practices, while the older children are likely free to select their own food, the means of parents’ or CPs’ practices on the feeding between the two age groups are different. FF, PK, and PBs interactively impact parents’ or CPs’ feeding practice of the younger children directly, but they facilitate the formation of healthy eating behaviors among the older children through parents’ or CPs’ advice or practical models guided by healthy FF (4–6, 20). Moreover, the parents’ or CPs’ and family characteristics also affect child feeding practice. A study revealed that male, lower literacy, and single-parent families were associated with unhealthy eating habits among the children aged 1–12 years (22). Similarly, our study showed that low FF score and male parent or CP were significantly associated with unfavorable PBs, while educational level, doing childcare by the parents, and larger family structure showed protective effects (Table 3). Normally, educational level impacts parents’ or CPs’ quality of childcare. Furthermore, childcare done by the parents is more effective than that done by the CPs. Therefore, corporate interaction among PK, PBs, parents’ or CPs’ characteristics, and family dynamics by FF is essential for the good nutritional health longitudinally. Many studies advocated to form healthy eating behaviors among the early childhood as they would be of benefit to their healthy nutritional status longitudinally (4, 6, 23–26). The designed intervention implemented for upgrading community children’s nutritional health and wellbeing requires an understanding of and a holistic approach to these related familial factors together.

## 5 Strengths and limitations

A limited sample size, participant reporting bias, that is, recall errors, social norms, or social standard-requirement biases, and inadequate child development data could confound the study results. Moreover, the generalizability of the results should be considered as differences in socio-economic status or culture-based practices between the study area and others, which may possibly affect. However, the study results indicated that not only FF, which was commonly attributed, but also PB, PK, and FCs were associated with child growth, especially during early childhood.

## 6 Conclusion

To improve the quality of child nurturing in the community, a thorough understanding of parental or CP and family factors is essential. Implementing effective nutrition management during early childhood or preschool age is very crucial, as such interventions can promote long-term healthy living.

## Data Availability

The original contributions presented in the study are included in the article/supplementary material, further inquiries can be directed to the corresponding author.
